# Effects of Aging on General and Specific Memory for Impressions

**DOI:** 10.1525/collabra.109

**Published:** 2018-05-11

**Authors:** Megan J. Limbert, Jennifer A. Coleman, Angela Gutchess

**Affiliations:** * Brandeis University, Waltham, Massachusetts, US; † Virginia Commonwealth University, Richmond, Virginia, US

**Keywords:** aging, memory, social memory, person memory, emotional memory

## Abstract

Despite the number of documented declines in memory with age, memory for socioemotional information can be preserved into older adulthood. These studies assessed whether memory for character information could be preserved with age, and how the general versus specific nature of the information tested affected outcomes. We hypothesized that memory for general impressions would be preserved with age, but that memory for specific details would be impaired. In two experiments, younger and older adults learned character information about individuals characterized as positive, neutral, or negative. Participants then retrieved general impressions and specific information for each individual. The testing conditions in Experiment 2 discouraged deliberate recall. In Experiment 1, we found that younger performed better than older adults on both general and specific memory measures. Although age differences in memory for specific information persisted in Experiment 2, we found that younger and older adults remembered general impressions to a similar extent when testing conditions encouraged the use of “gut impressions” rather than deliberate retrieval from memory. We conclude that aging affects memory for specific character information, but memory for general impressions can be age-equivalent. Furthermore, there is no evidence for a positivity bias or differences in the effects of valence on memory across the age groups.

Despite a large body of research documenting memory declines with age (Park et al., 2002; Salthouse, 1996; Zacks, Hasher, & Li, 2000), recent work suggests exceptions to the pattern of age-related decline in socioemotional domains, depending on the positivity, motivational relevance, or importance to the self of information particularly in socioemotional domains (as discussed in Carstensen & Mikels, 2005; Hess, 2005; Kensinger & Gutchess, 2015). In these studies, we investigate whether the demands to remember specific details as opposed to general information can contribute to the occurrence and magnitude of age differences, with a focus on memory for the character of others.

Despite the growth in research at the intersection of motivation and cognition (e.g., Madan, 2017), there has been relatively little research explicitly addressing the effects of aging on memory for information about one’s character (e.g., is this person “good” or “bad”?). This is an important question to address, in that older adults may make many decisions based on character information, such as who to trust with financial investments or which medical professional to recommend to a friend. If older adults’ memory for impressions and character information is impaired, even at this general level, it would indicate that decision aids and support may be needed with such decisions. Should it be intact, that may suggest that older adults should have confidence in their memory and decisions based on such information, perhaps even more than other memory domains. Some evidence indicates that impression memory may be relatively spared with aging. Older adults may remember their impressions of others to the same extent as younger adults (Todorov & Olson, 2008), though their sample was relatively middle-aged, with an average age of 57. Subsequent work with an older sample converges with those results to show that memory for trait information (e.g., rude, curious) can be age-equivalent, but only when information is framed in a personally meaningful way (Cassidy & Gutchess, 2012). Meaningful goals seem to effectively engage older adults’ cognitive resources, increasing task performance (Hess, 2006; Hess, Follett, & McGee, 1998; Hess, Germain, Swaim, & Osowski, 2009). Thus, older and younger adults may remember different character information that is in line with their goals. For example, inconsistent behaviors are more difficult for older adults to remember than young, because younger adults spontaneously attempt to explain inconsistencies in behavior (Hess & Tate, 1991). Character information has been found to serve as a beneficial mediator of face-name memory for both younger and older adults, hinting at its potential value in memory (Old & Naveh-Benjamin, 2012).

The distinction between memory for character information and other types of memory is also illustrated by work with patient groups, which demonstrates that character information can be retained despite memory impairments. Patients with amnesia due to Korsakoff’s syndrome adequately encode character information such that they correctly express a preference for a target individual associated with “good” characteristics over a target individual associated with “bad” characteristics (Johnson, Kim, & Risse, 1985). This learning of general character information occurs in the absence of memory for the specific supporting details regarding character. Results from another study converged. An amnesic individual with hippocampal damage remembered face-trait associations to the same extent as healthy controls when tested with a forced choice recognition test (i.e., which of these two people is the nicer individual?) (Todorov & Olson, 2008). In the same study, patients with damage extending into the amygdala and temporal pole exhibited impaired memory for character information. These findings suggest that the neural system supporting character memory is separable from the system supporting other types of explicit memory. Given the effects of Korsakoff’s syndrome and hippocampal damage on memory, individuals with less severe memory impairments, including older adults, should be able to accurately encode interpersonally relevant character information, at least at the general level of impressions.

Although older adults can remember information as well as younger adults when information is relevant and meaningful to one’s life (Cassidy & Gutchess, 2012; May, Rahhal, Berry, & Leighton, 2005; e.g., Rahhal, May, & Hasher, 2002; American participants in Yang, Chen, Ng, & Fu, 2013), it may be that, as is the case with amnesic patients (Johnson, Kim, & Risse, 1985; Todorov & Olson, 2008), the effects operate at a general level and do not improve memory for specific information. When forming impressions, older adults are less likely to use specific trait information in their organization of information (Hess et al., 1998) and have more difficulty remembering inconsistent information than younger adults (Hess & Tate, 1991); access to that specific information is particularly demanding of cognitive resources (Hess, 2006; Hess et al., 2009). In the broader memory literature, there is a large body of work showing that older adults emphasize gist, or general memory, but exhibit impaired memory for specific details (e.g., Koutstaal & Schacter, 1997; Tun, Wingfield, Rosen, & Blanchard, 1998; though see Koutstaal, 2006 for evidence of retrieval flexibility for younger adults and, to some extent, older adults). For example, older adults are accurate at remembering the general range of prices and which grocery store item is a “better buy” (Castel et al., 2005; Flores, Hargis, McGillivray, Friedman, & Castel, 2017), indicating intact memory for gist for another type of everyday information. Thus, the distinction between general and specific levels of memory may prove useful for socioemotional domains, such as remembering impressions of others.

Memory for emotional information has shown the importance of the distinction between general and specific memory. Negative arousing information can improve memory for specific visual details (e.g., which exemplar of a gun was studied previously?) in younger adults (Kensinger, Garoff-Eaton, & Schacter, 2006) as well as in older adults (Kensinger, Garoff-Eaton, & Schacter, 2007). Emotional valence (i.e., the positivity or negativity of information), however, had different effects on general memory with age. Younger and older adults’ general memory was superior for negatively valenced information, compared to neutral information, although positively valenced information also benefited older adults’ general memory. Denburg and colleagues also find a distinction between general and specific information. Their work suggests that emotion helps younger and older adults to encode the gist, or general theme of information, but does not support the encoding of specific details (Denburg et al., 2003). These studies illustrate that the distinction between general and specific levels of memory is important for the study of emotional memory.

Recent neuroimaging work also indicates a dissociation between the neural regions implicated in general vs. specific memory for socioemotional information. In an fMRI study by Somerville, Wig, Whalen, & Kelley (2006), faces were paired with information in order to color the character of an individual as positive, negative, or neutral. The participants’ task was to determine whether they had seen the person’s face before and to recall any specific information about that person (their name or information from the sentence). The authors found that the right hippocampus responded to all faces paired with a description regardless of valence, but that amygdala activation occurred during remembering general impressions of faces that had been paired with valenced (good or bad) information; this activation was independent of whether the individual could remember any detailed information related to the stimuli’s valence. These results suggest a dissociation between general and specific memories, with memory for general socioemotional information supported by the amygdala.

In the present two studies, we investigate the effects of aging on general and specific memory for character information. Although previous studies have examined memory for character information, these investigate young adults (Somerville et al., 2006) and amnesic patients (Johnson et al., 1985; Todorov & Olson, 2008). We extend this body of work to healthy older adults, and also explicitly investigate memory across levels, examining the effects of aging on general memory for impressions of others as “good” or “bad” as well as memory for details, drawing on a distinction that has proved useful in non-social domains of memory (e.g., Koutstaal & Schacter, 1997). We predict that socioemotional conditions can benefit memory accuracy at the general level of whether something is “good” or “bad”. However, we predict that socioemotionality will disproportionately benefit general rather than specific memory for older adults such that younger and older adults will retrieve similar amounts of general information, but younger adults will retrieve more specific details upon which impressions were based.

As character impressions are considered as “good” or “bad”, we furthermore explicitly investigate how aging impacts memory for the different levels of valence. This connects with a rich, but varied, literature in the domain of emotion and age. Although older adults sometimes exhibit a positivity effect, remembering relatively more positive information than young (e.g., Charles, Mather, & Carstensen, 2003; Mather & Carstensen, 2005; Reed, Chan, & Mikels, 2014), this finding is not pervasive (see Murphy & Isaacowitz, 2008). In some cases, negative arousing information is most beneficial for older adults’ memory (Kensinger et al., 2007). There is some evidence that memory for character information could be influenced by aging such that younger adults better remember negative information whereas older adults tend to better remember positive information (e.g., Leshikar, Park, & Gutchess, 2015) and engage neural regions in line with this age difference (Cassidy, Leshikar, Shih, Aizenman, & Gutchess, 2013). However, the inconsistency of effects of valence on memory for impressions with age (e.g., Cassidy & Gutchess, 2012) across the few studies on this topic did not support clear predictions. The present experiments allow us to conduct exploratory analyses to assess the contribution of valence to memory for character information.

## Experiment 1

### Methods

#### Participants

Twenty students, ranging in age from 18–22 (*M* age = 18.95, *SD* = 1.15; 11 females), from Brandeis University and twenty healthy older adults, ranging in age from 61–88 (*M* age = 75.15, *SD* = 6.29; 11 females), from the greater Boston area participated for course credit or pay. Sample size was determined based on other related studies (e.g., Todorov & Olson, 2008); according to calculations with G*power, detecting an interaction between age and learning of valenced trait information would require n = 14 per group to detect effects at an effect size of f = .29. Older adults had scores of 26 or above on the Mini-Mental State Exam (MMSE) (Folstein, Folstein, & McHugh, 1975). Data from one additional older adult were excluded due to failure to meet the criteria.

#### Stimuli

**Figure 1:**
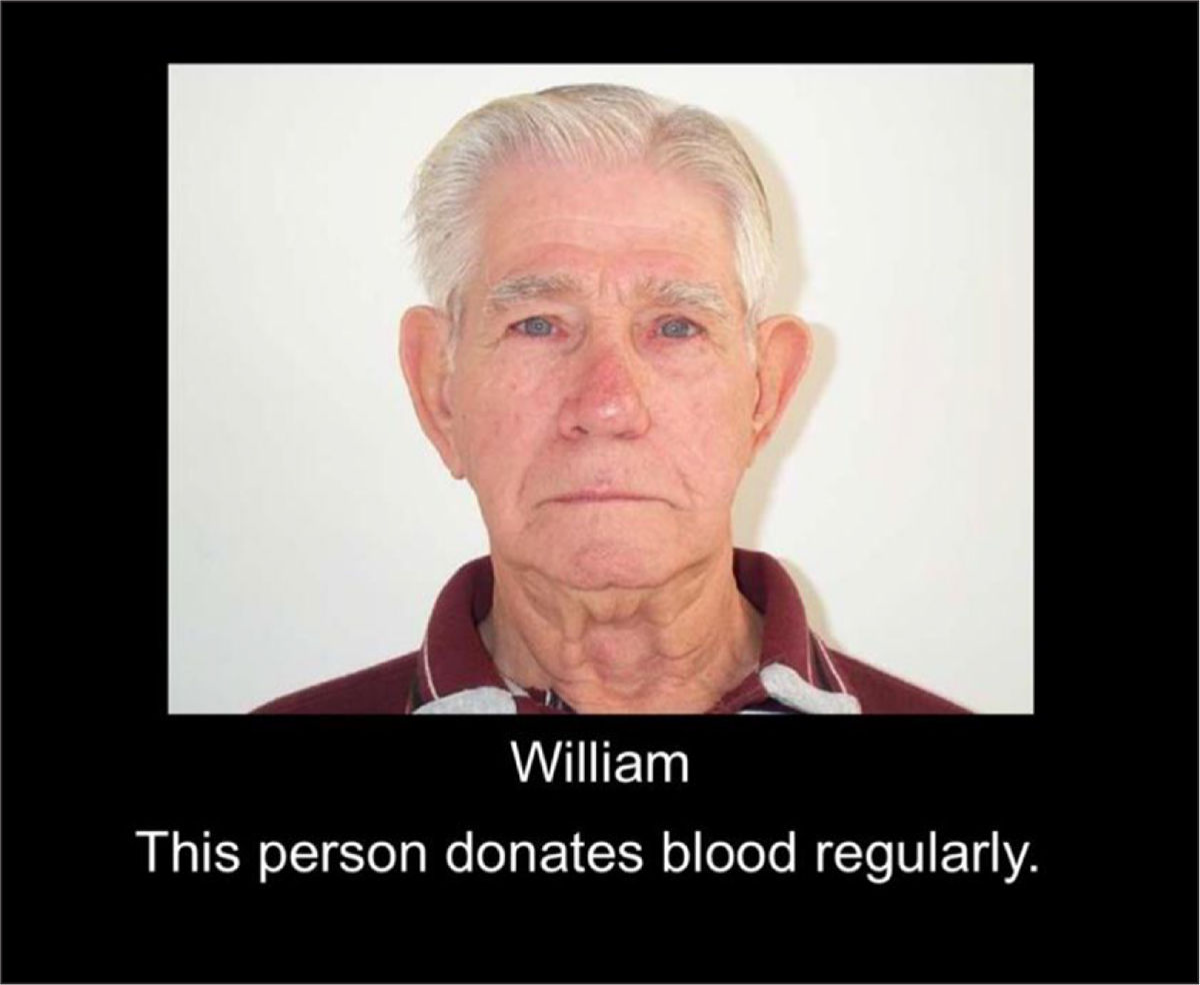
Example stimulus consisting of a face, name, and sentence indicating positive behavior. Actual stimuli were presented in color.

Participants passively viewed 48 different faces presented one at a time on a computer. As shown in Figure 1, faces were presented with a name and a sentence providing contextual information about them, such as, “This person saved someone’s life”, “This person uses blue pens”, or “This person is a murderer”. The sentences manipulated the valence of the character information (good, neutral, or bad).

##### Faces.

Face stimuli were obtained from the Productive Aging Lab at the University of Texas at Dallas database (https://pal.utdallas.edu/facedb/). Forty-eight faces were selected based on perceived ages (ranges 18–24, *M* = 22.7 years and 60–69, *M* = 64.1 years) using equal numbers of younger and older and male and female faces. We selected faces based on norms (Kennedy, Hope, & Raz, 2009) that allowed us to equate faces on the dimensions of familiarity, memorability, mood, and picture quality across the gender and age groups (*F*s < 2.1).

##### Names.

The names were selected from the United States Social Security Administration’s database of most popular baby names for given years (http://ssa.gov/OACT/babynames). After eliminating duplicate and similar (e.g. Christopher and Christine) names among age and sex groups and the name of an experimenter, 12 of the top 20 most popular names for males and females for the years 1943 and 1985 were collected for random assignment to age- and gender-congruent “old” and “young” faces. These two years were selected as the years the stimulus individuals would have been born based on the average perceived ages for the face stimuli for each age group.

##### Sentences.

The sentences that were presented with the faces in order to create a good, bad, or neutral context were a subset of those used by Somerville et al. (2006). Analyses for the ratings of valence and arousal obtained from twenty-three younger adults in their study were combined with pilot ratings of six older adults in order to select the most appropriate sentences for the present study. We excluded sentences with *SD* > 1.5 for valence ratings and *SD* > 2.0 for arousal ratings in order to select stimuli that were rated most similarly across participants. To ensure that positive and negative sentences did not differ on arousal, the remaining positive and negative sentences were then matched for arousal (mean ratings from 4.0–5.9, on a scale of 1–9, 9 being most arousing) and sentences with the most extreme positive and negative valence ratings within this subgroup were selected. There were no significant differences in arousal (*t* = 1.85, *p* < .08) or distance from a neutral valence (*t* = .71, *p* = .48) for positive and negative sentences. Neutral sentences were chosen by selecting the least arousing sentences and then selecting those sentences with valence ratings the closest to zero after eliminating sentences with highly variable valence ratings (*SD* > 2.0), (arousal *M* = 1.68, *SD* = 1.21; valence *M* = .13, *SD* = 0.59 on a scale of −4 to 4, −4 being most negative, 4 being most positive). Sentences were assigned to the 48 face-name pairs, with equal numbers of good, bad, and neutral behaviors randomly assigned to faces of each age and gender. Four different combinations of stimuli (face/name/sentence) were created for randomly-assigned use during the encoding phase. During this encoding phase, participants were presented with 16 face/name/sentence combinations of each context (good, bad, or neutral). The presentation of the stimuli was pseudo-randomized. An online randomization program (http://random.org/lists/) was used to determine the order, but the order was then manually corrected so that there were no more than 3 sentences of one valence, 4 faces of one age, or 4 faces of one sex presented sequentially.

#### Procedure

Participants provided written informed consent for the study, approved by the Brandeis University Institutional Review Board. Next, participants received instructions for encoding: “When you are viewing the slides, imagine you are meeting the individual for the first time. Read and try to learn the information about them. What is this person’s name? Based on the information presented about them, what type of impression do you form about this person?”. The face-name-sentence triads each were presented for 5 seconds, with a blank screen for 1 second before the next triad. After all 48 triads were presented, they were repeated again two more times in new orders so that all triads were seen a total of three times. The experiment was conducted with E-Prime 2.0 (Psychology Software Tools, Pittsburgh, PA).

For 30 seconds after encoding, participants counted backwards by 7 in order to eliminate recency effects in the following memory task. Participants were then presented with only the faces and names of each previously-learned character in a random order and were asked 1) what kind of impression they formed of this person and, 2) if they could recall any additional information about them. Participants made a keypress to indicate their positive, neutral, or negative assessment of the individual and reported aloud their recall of any additional character information. An experimenter recorded these verbal responses for later scoring.

Additional neuropsychological tests were then administered to characterize our samples, with results presented in Table 1. Participants completed demographics and health questionnaires, Verbal Paired Associates I to assess associative memory (Wechsler Memory Scale – III, Wechsler, 1997), a digit comparison task to assess speed of processing (Hedden et al., 2002; modeled after Salthouse & Babcock, 1991 Letter Comparison Task), a letter-number sequencing task to assess working memory and executive function (Wechsler Memory Scale – III, Wechsler, 1997), and the Shipley Vocabulary Test to assess vocabulary as a type of crystallized intelligence (Shipley, 1986). Older adults completed the MMSE (Folstein et al., 1975).

#### Scoring Specific Memory

The verbal responses to the question of, “What other information do you remember about this person?” were coded using a scheme that allowed the main idea to be communicated through rephrasing and/or synonyms (e.g. for a target of “helps the elderly”, “likes old people” was accepted; for a target of “embezzler”, “steals money”, “is a crook”, or “writes bad checks” were accepted). Two individuals separately scored each participant’s responses. The Krippendorff’s alpha statistic for inter-rater reliability was .99. In order to have only one score for each participant, any discordance between the two judges’ scores was resolved through discussion with an additional member of the research team.

### Results

#### General Memory

The ability to remember general, gist-based character information was measured as the number of correct responses to the question, “What kind of impression did you form of this person?”, that was presented during the recognition trial, with the option of responding with “positive”, “neutral”, or “negative”. In order to correct for potential group differences in guessing biases (i.e., the tendency to use “positive”, “neutral”, or “negative” labels), we used the adaptation of Cohen’s (1960) kappa statistic, as devised by Isaacowitz et al. (2007). It is important to use a measure of corrected recognition for our general memory data in order to distinguish the ability to correctly discriminate information in memory from response bias. A participant, for example, could tend to predominantly choose the “positive” response option. This may lead to the appearance of the excellent performance in the positive trials, but it would not account for all of the times that the “positive” response was misapplied to other conditions. Using kappa scores allows for a more appropriate comparison of memory sensitivity across conditions and age groups, whereas raw scores could reflect the bias to use different labels. Kappa is calculated as k = (number of correct responses – number of chance-expected correct responses)/(the total number of items – number of chance-expected correct responses). Kappa scores can range from 1 for perfect classification performance to 0 for chance performance (or below 0 when classification performance is below chance).

**Figure 2:**
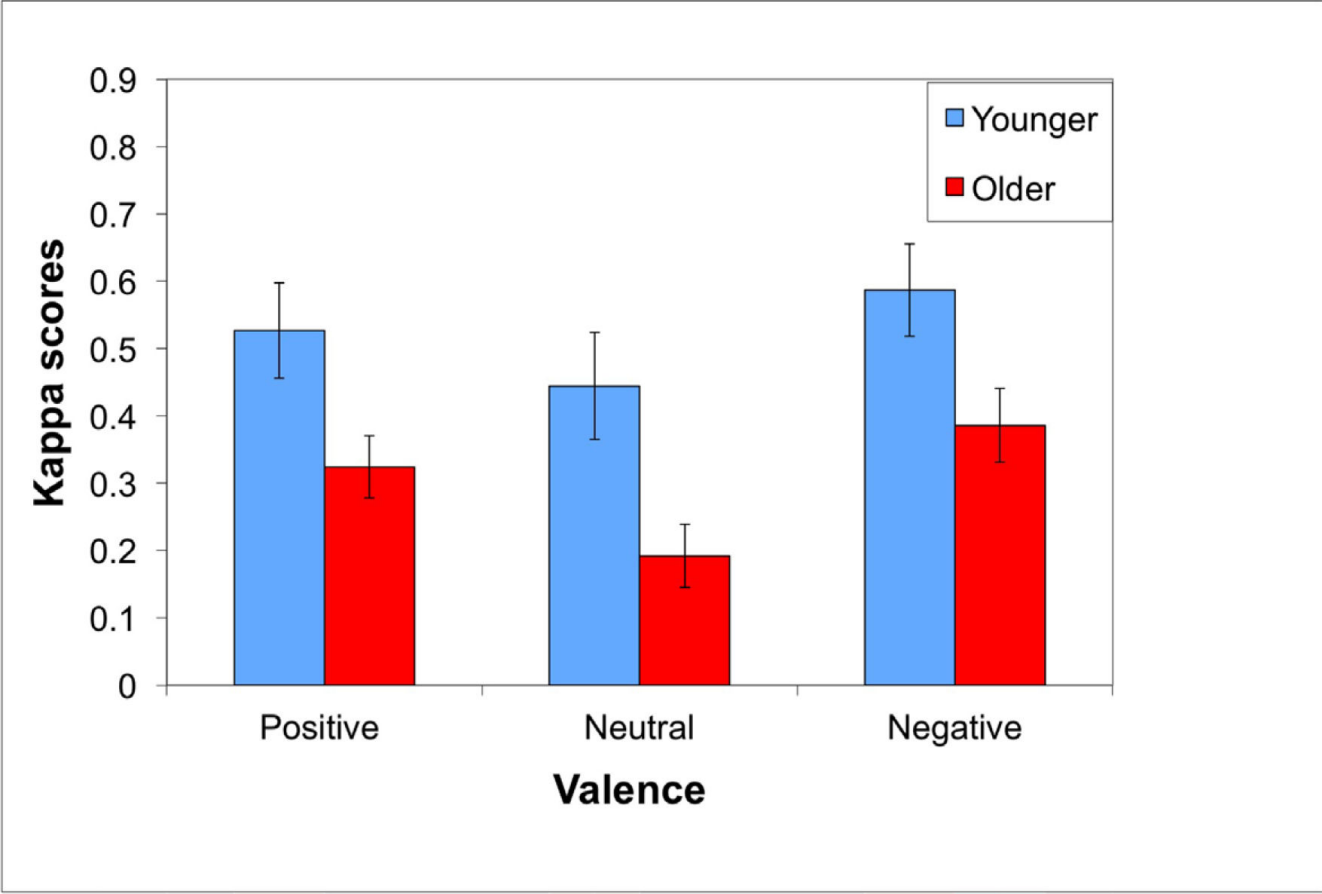
In Experiment 1, average kappa-corrected general memory scores (+SE) were higher for younger than older adults. Scores were higher for negative stimuli compared to positive, which were higher than neutral stimuli.

Kappa scores were analyzed in a 2 × 3 mixed design ANOVA, with age (younger/older) as the between participants variable and valence (positive/neutral/negative) as the within-participant variable. As shown in Figure 2, results revealed significant main effects of age, *F*(1, 38) = 6.99, *p* = .01, η_p_^2^ = .16, and valence, *F*(2, 76) = 20.86, *p* < .001, η_p_^2^ = .35, but no age by valence interaction, *F*(2, 76) = .60, *p* = .55, η_p_^2^ = .02. Younger adults performed better than the older adults, negative information was retrieved significantly better than positive, *t*(39) = 2.24, *p* = .03, and positive information was retrieved significantly better than neutral, *t*(39) = 5.24, *p* < .001.

#### General Memory Reaction Times

Reaction times for correct general memory trials were analyzed in a 2 × 3 mixed design ANOVA, with age (younger/older) as the between participants variable and valence (positive/neutral/negative) as the within participant variable. There was a significant main effect of age, such that older adults were slower than younger adults, *F*(1, 35) = 17.42, *p* < .001, η_p_^2^ = .33. The main effect of valence was also significant, *F*(2, 70) = 5.06, *p* = .009, η_p_^2^ = .13, whereas the interaction of valence × age was not: *F*(2, 35) = 2.05, *p* = .14, η_p_^2^ = .06. As shown in Table 2, follow-up paired t-tests revealed that reaction times for correct positive trials were faster than for neutral trials, *t*(36) = 2.58, *p* = .014, and there was a trend for positive to be faster than negative, *t*(36) = 1.97, *p* = .06. Reaction times to negative and neutral trials did not significantly differ from each other, *t*(36) = .94, *p* = .36.

#### Specific Memory

Scores for specific memory performance were determined for each individual by first eliminating trials for which general impressions were *not* recalled correctly. This meant that of the 16 items presented for each condition, on average between 10.40–11.55 trials were scored for each condition for specific memory for younger adults and between 7.45–10.85 trials for older adults. The specific memory hits were converted into proportions indicating the percentage of the correct general impressions for which specific information was also remembered, which accounted for potential age and individual differences in the amount of general information remembered. This was done for each level of valence.

**Figure 3:**
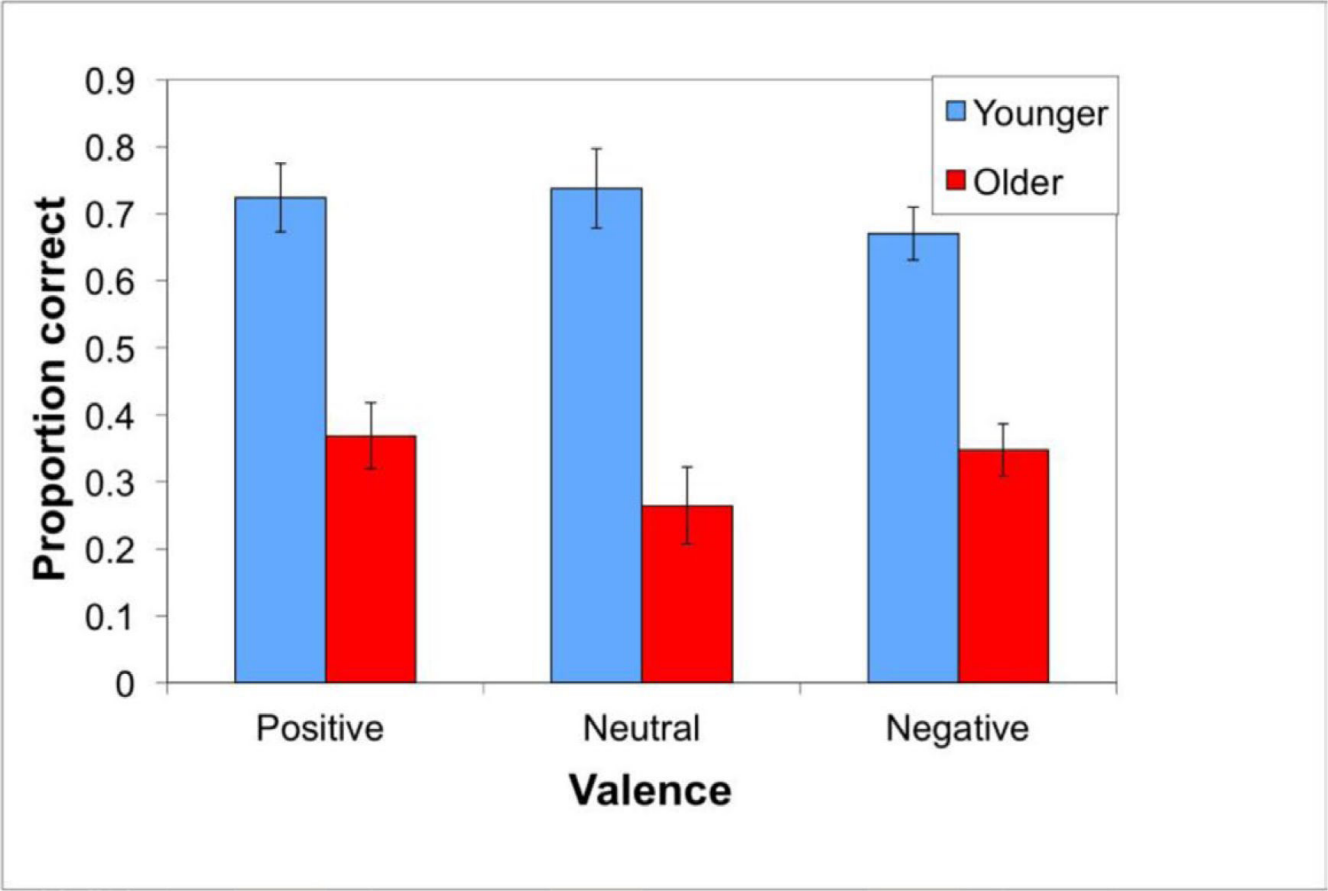
Specific memory scores for Experiment 1 are higher for younger than older adults, with a trend towards older adults performing worse on neutral than positive trials, whereas younger adults did not differ across conditions. The graph depicts average performance (+SE) on the specific memory task.

We conducted a 2 × 3 mixed-design ANOVA on the specific memory scores, with age group as the between participant and valence as the within-participant variables. As shown in Figure 3, there was a significant main effect of age, *F*(1, 38) = 44.02, *p* < .001, η_p_^2^ = .54, with younger adults outperforming the older adults, but no significant effect of valence, *F*(2, 76) = .94, *p* < .40, η_p_^2^ = .02. There was also a marginal age by valence interaction, *F*(2, 76) = 2.53, *p* = .09, η_p_^2^ = .06. This trend is driven by the neutral items, with older adults scoring worse on neutral than positive items, *t*(19) = 2.22, *p* = .04, whereas younger adults did not differ across levels of valence (all *t*s < 1.6).

### Discussion

The results of Experiment 1 indicate that both general and specific memory for character information are impaired with age. In addition, a positivity bias did not emerge for older adults in the accuracy of memory for character information. Across younger and older adults, general memory for valenced information (negative and positive) is higher than for neutral information, with the highest levels of memory for negative information. Response times are fastest for positive general memory trials, but only statistically so compared to neutral trials. For specific memory, although there is a trend for older adults to better remember positive than neutral information, this does not emerge strongly. Importantly, no differences in specific memory emerge for positive versus negative valence, for younger or older adults. These results will be discussed in turn.

Based on previous studies in which socioemotional or personally meaningful content eliminated age differences in memory (e.g., Cassidy & Gutchess, 2012; May et al., 2005; Rahhal et al., 2002; American participants in Yang et al., 2013), we had predicted that older adults would perform as well as younger adults in remembering general impressions of others, but not the specific details of behaviors. These predictions were also informed by findings with amnesic patients that despite being unable to remember specific reasons for feeling a certain way towards someone, patients’ ability to remember general impressions of individuals was spared (Johnson et al., 1985; see also Todorov & Olson, 2008). In fact, our results show that older adults did not remember either specific information or general impressions as well as younger adults.

Aspects of our design may have contributed to the similar pattern of age deficits across specific and general memory. In our procedure, there was an expected sequence of events in that participants reported their positive, negative, or neutral impression of one person, followed immediately by recalling any additional details for that individual. We expect that while contemplating the first question, participants were also thinking of the second question and basing their answer on their explicit memory for specific details. If all the general impressions were to be elicited before probing memory for specific details, we may be able to disentangle the effects of aging on general and specific memory. This will be assessed in Experiment 2.

Additionally, we found no evidence for a positivity bias when using kappa scores to compared corrected memory scores, corrected for guessing biases (based on Isaacowitz et al., 2007). Socioemotional selectivity theory (Carstensen, Isaacowitz, & Charles, 1999) predicts that older adults who are better emotion regulators than younger adults can, in some instances (e.g., Charles et al., 2003; Mikels, Larkin, Reuter-Lorenz, & Carstensen, 2005; Reed et al., 2014), remember positive information better than neutral or negative information (but see Murphy & Isaacowitz, 2008). It is possible that in memory for character information, there is no advantage for positive information or that this bias only emerges when behaviors have implications for the person encoding the information in memory. Some prior work highlights the importance of goals or contextual factors in age differences in cognitive processes, including memory for impressions of others (e.g., Cassidy & Gutchess, 2012; Hess, 2006; Hess et al., 1998; Hess et al., 2009; Leshikar et al., 2015). The present task did not have a strong framing around personal or motivational goals, which could have prevented the emergence of valence effects. However, it is also possible that positivity effects emerge more consistently for arousing or valenced information that lacks social implications, in that both positive and negative behaviors of others have implications for social goals.

One limitation of Experiment 1 is that as the measures were designed, specific and general memory performance may have been closely coupled. As previously noted, because general and specific memory were assessed in succession for each face, participants may have attempted to consciously retrieve information from memory rather than relying on their gut impressions formed implicitly from prior experiences (as was the case for the amnesic patients tested by Johnson et al., 1985 and Torodov & Olson, 2008). We address these concerns in Experiment 2.

## Experiment 2

In Experiment 1, older adults performed worse than younger adults on measures of both general and specific memory. By emphasizing the importance of relying on a gut impression, adapting the format of the general memory test, and separating general and specific memory tests to be administered at different points in time, we aimed to reduce participants’ potential tendency to rely on explicit memory in Experiment 2, thus better simulating conditions along the lines of prior work with amnesics (Johnson et al., 1985; Todorov & Olson, 2008).

### Methods

#### Participants

Twenty-eight students, ranging in age from 17–25 (parental consent provided for those aged 17; *M* age = 18.93, *SD* = 1.46; 23 females), from Brandeis University and twenty-eight older adults, ranging in age from 59–91^1^ (*M* age = 72.19, *SD* = 8.83; 22 females) from the greater Boston area were recruited using criteria identical to Experiment 1.^2^ One additional younger and one additional older participant were eliminated from the sample because they scored at chance on the general memory task, eight additional young adults were discarded due to administration errors, and one younger adult was eliminated to match the sample size for older adults. See Table 1 for sample characteristics.

#### Procedure

The stimuli and procedure were the same as those for Experiment 1, with the following changes. The 48 face-name-sentence triads were presented for only two runs. After the 30 second retention interval (i.e., counting backwards by 7s), the two memory tasks were administered separately, in order to better distinguish general and specific memory. In the first test of general memory, participants saw two faces side by side and were asked to decide which face they felt more positive about. They were instructed to rely on their gut impression rather than recalling past learned information. Participants responded by pressing one of two keys to indicate which face they felt more positive about. All 48 faces were shown, and the faces in the 24 pairs were matched by gender (i.e., two females or two males) and age (i.e., two younger or two older). The pairings were presented in a random order and four different versions of the task were created based on the 4 different encoding versions. Pairs were assigned to conditions of a positive face and a negative face (Pos/Neg), a positive face and a neutral face (Pos/Neu), or a neutral face and a negative face (Neu/Neg). Similarly, corrections were made to prevent face/name/behavior triads from being placed in the same condition in all four counterbalanced versions. The specific recognition task followed the completion of the general memory test. The specific recognition test and neuropsychological tests were identical to those used in Experiment 1.

### Results

#### General Memory

**Figure 4:**
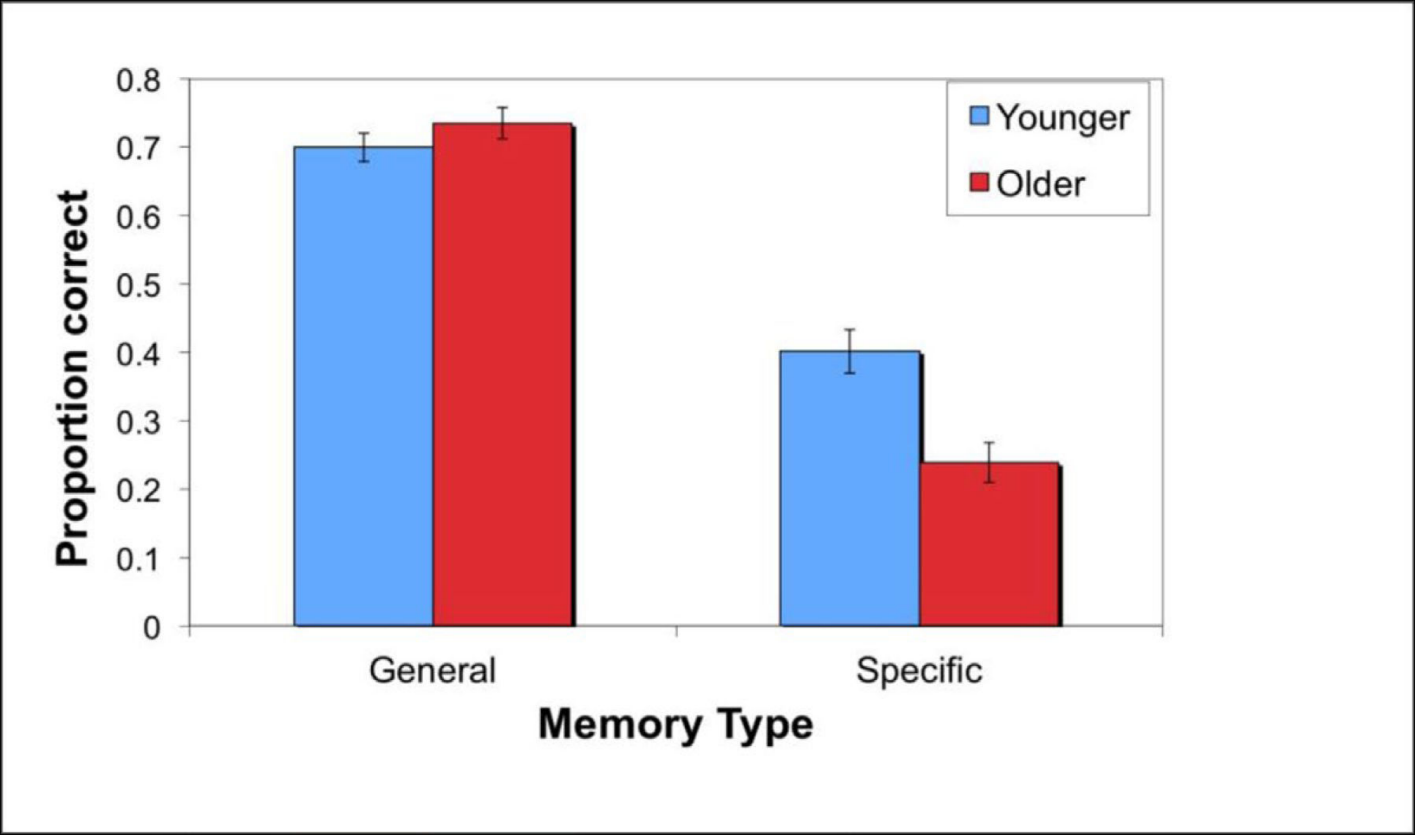
In Experiment 2, younger and older adults remembered general impressions equivalently, whereas young adults remembered more specific character information than older adults. The graph depicts average performance (+SE) on general and specific memory tasks, collapsed across valences.

For general memory, we conducted a 2 × 3 mixed design ANOVA with age (younger/older) as the between participants variable and valence (negative vs. neutral, positive vs. neutral, positive vs. negative) as the within-participant variable. There was a significant main effect of valence, *F*(2, 108) = 16.48, *p* < .001, η_p_^2^ = .23, with follow up tests revealing that relative to the comparison of positive vs. neutral faces (*M* = .62, *SD* = .17), participants were more accurate at selecting the positive face vs. the negative one (*M* = .79, *SD* = .18), *t*(55) = 5.63, *p* < .001, and the neutral face vs. the negative one (*M* = .75, *SD* = .18), *t*(55) = 3.67, *p* = .001. The main effect of age, *F*(1, 54) = 1.30, *p* = .26, η_p_^2^ = .02, and the age × valence interaction, *F*(2, 108) = .52, *p* = .60, η_p_^2^ = .01, did not approach significance. See results in Table 3 and Figure 4.

#### General Memory Reaction Times

Reaction times to correct trials were analyzed. Although there was a main effect of age such that younger adults were faster than older adults, *F*(1, 54) = 16.96, *p* < .001, η_p_^2^ = .24, the main effect of valence, *F*(2, 108) = 2.20, *p* = .12, η_p_^2^ = .04, and the interaction of age × valence were not significant, *F*(2, 108) = .31, *p* = .74, η_p_^2^ = .01.

#### Specific Memory

For specific memory, we conducted a 2 × 3 mixed design ANOVA with age (younger/older) as the between-participants variable and valence (negative, neutral, positive) as the within-participant variable. As shown in Table 3, there was a significant main effect of valence, *F*(2, 108) = 6.60, *p* = .002, η_p_^2^ = .11, with specific memory for positive items (*M* = .28, *SD* = .19) worse than memory for negative (*M* = .33, *SD* = .19), *t*(55) = 2.76, *p* = .008, and neutral (*M* = .35, *SD* = .21) items, *t*(55), 3.77, *p* < .001. The main effect of age was also significant, *F*(1, 54) = 14.44, *p* < .001, η_p_^2^ = .21, with younger adults (*M* = .40, *SD* = .17) outperforming older adults (*M* = .24, *SD* = .15), but the age × valence interaction was not significant, *F*(2, 108) = .42, *p* = .66, η_p_^2^ = .01.

#### Comparison of Reaction Times across Experiments 1 and 2

In addition, we conducted exploratory analyses of reaction times for general recognition across the two experiments.^3^ These analyses were performed post hoc in an attempt to substantiate our claims that the general recognition test in Experiment 2 relied on more automatic processes than the general recognition test in Experiment 1, perhaps particularly benefiting older adults. As shown in Table 2, although both age groups respond faster on the general memory task in Experiment 2, the facilitation is larger for older than younger adults. This is seen in reaction times that are nearly half the duration in Experiment 2 compared to Experiment 1 in younger adults, but older adults show an even greater facilitation. A 2 × 2 × 3 mixed ANOVA, conducted across the two experiments, with age and valence as the additional factors, did reveal a significant age × study interaction, *F*(1, 89) = 7.11, *p* = .01, η_p_^2^ = .07. A comparison of the effect sizes for the age effect in Experiment 1 (η_p_^2^ = .33) with Experiment 2 (η_p_^2^ = .24) is consistent with the idea that emphasizing more automatic, rather than deliberative memory decisions, in Experiment 2 facilitates general memory performance, particularly for older adults. The main effects of study, *F*(1, 89) = 49.83, *p* < .001, η_p_^2^ = .36, and age, *F*(1, 89) = 37.77, *p* < .001, η_p_^2^ = .30, were also significant. We only report the effects of age and study here, in keeping with our interest in using reaction times to substantiate the different types of decisions made across the two experiments, but it is important to note that the effects of valence would not be particularly interpretable as the valence levels were not tested as independently in Experiment 2 (i.e., participants decided which of two faces was more positive and faces were selected from two different levels of valence).

### Discussion

The results of Experiment 2 indicate that memory for general memory for impression information can be intact with age, even when memory for specific information about others is impaired. Our findings could account for the age-equivalent memory for information regarding trustworthiness, character, or safety (May et al., 2005; Rahhal et al., 2002; American participants in Yang et al., 2013), in that only a general impression (e.g., “good” or “bad”) was required in those tasks. It may be that memory for socioemotionally relevant information is preserved with age to some extent, but not enough to support memory for specific, detailed information. Older adults tend to rely less on this type of detailed information (Hess et al., 1998), likely because it requires additional cognitive resources and does not support the motivational goals (Hess, 2006; Hess et al., 2009). Moreover, this finding is consistent with prior work on memory for emotional information in that older adults’ memory for specific details is less enhanced by emotional content than younger adults’ (Denburg, Buchanan, Tranel, & Adolphs, 2003).

In terms of valence, we again find no evidence of a positivity bias in memory for character information, and no differences across the age groups in the valence of material that tended to be remembered, for general or specific memory. In fact, specific memory for positive information tended to be worse in this experiment. Although this could suggest that it is more advantageous to remember negative information about others across the lifespan, it also could reflect unintended differences in the types of character information presented in each condition. Future studies could better address this question by using stimuli that are more equivalent across the different types of valence, such as trait words. In addition, it is important to note that the general memory task, in which the participant selected which of two faces was more positive (based on the prior pairing of the faces with sentences), did not allow for a clean and direct comparison of the different levels of valences. Furthermore, the nature of the test may have biased memory scores based on the properties of the lure face. For example, memory tended to be better for distinguishing positive or neutral faces from negative ones, whereas memory for positive and neutral faces did not differ from each other. The orientation to selecting the positive face may have been most salient when in contrast with the most negative information.

## General Discussion

Across two studies, we find some evidence that age may impact memory for character information, in that specific memory may be poorer in older adults than general memory. However, this finding depends on task demands. Older adults were impaired on both types of memory in Experiment 1, when participants may have attempted to rely on more explicit memory for previously learned character information. When put in a situation in Experiment 2 that discouraged a search of explicit memory, older adults’ general memory performance reached, or even slightly exceeded, the level of younger adults’ performance. This pattern is consistent with the idea of two different routes to support memory. Traditionally this has been thought of as the distinction between explicit (e.g., conscious) memory and implicit (e.g., nonconscious) memory, with suggestions that explicit memory may undergo more age-related decline than implicit memory (Light & Singh, 1987). Older adults may default to using their explicit system for memory tasks, perhaps reflecting their familiarity with approaching memory tasks in this manner and a lack of metacognition about other potential strategies or the systems that are relatively more preserved with age. This reliance on explicit systems may occur even though relying on their more intact implicit system could be more effective for retrieving general impressions about one’s character. By discouraging the use of explicit memory in Experiment 2, we may have helped older adults to draw on this more intact system and set of information about general impressions. We discuss potential neural correlates of these systems in the next paragraph. The present finding may help to unite literatures documenting extensive decline in memory with age (as reviewed by Zacks et al., 2000) with the surprising newer work revealing age-equivalent memory for socioemotional information (May et al., 2005; Rahhal et al., 2002). The current studies suggest that age impairments in memory for social information depend on the necessity of retrieving general versus specific memory, and having a context that supports implicit affective-based responding (see Cassidy & Gutchess, 2012, for a related demonstration of the effects of task orientation on memory for socioemotional information).

In terms of relevant neural systems, the intact performance on general memory in Experiment 2 is consistent with literature suggesting that amnesia does not impair memory for character information (Johnson et al., 1985; Todorov & Olson, 2008) and that the amygdala, rather than the hippocampus, subserves encoding of character information that is consistent with impressions formed (Schiller, Freeman, Mitchell, Uleman, & Phelps, 2009). Thus, it may be the case that an amygdala-based system supports memory for general impressions in a manner similar to implicit memory. Neuroimaging methods would allow for a test of these ideas in healthy older adults. Functional changes in the brain regions involved in socioemotional processes have been little-investigated in older adults, with most research examining the effects of emotion per se (see Kensinger & Gutchess, 2015 for a review). With this task, we would predict a dissociation between the neural networks that respond to valenced and non-valenced information, and specific and general memory, consistent with the results of Somerville et al. (2006). More specifically, we expect that general information would be supported by systems involved in the automatic processing of information (e.g., amygdala) and that specific information would be supported by systems governing controlled processing (e.g., hippocampus, prefrontal cortex), which are more impacted by the aging process. Research in this area would help to discern the extent to which socially-relevant information is preserved with age due to its reliance on distinct neural systems than memory for relatively neutral information, at least for general memory.

Although much of the research thus far has focused on how age-related changes in motivation affect memory for emotional information (e.g., Charles et al., 2003; Fung & Carstensen, 2003; Mather & Carstensen, 2003), we do not believe that emotionality solely accounts for the prioritization of social information in memory. Both positive and negative information about others can be relevant to goals, and both types could be useful in different social situations. For example, perhaps an aggressive or assertive individual is someone you would want as a teammate or legal advocate, whereas a caring individual is someone you would want as a friend or caregiver when recuperating from surgery. Other work is consistent with our findings in showing that there is not an overall bias for positive or negative character information to be better remembered by younger or older adults, but that additional factors, such as whether the trait is shared by the self (Leshikar, Cassidy, & Gutchess, 2015; Leshikar & Gutchess, 2015; Leshikar, Park, et al., 2015) or pertains to morality (Hess & Kotter-Grühn, 2011) can influence whether positive or negative trait information about others is prioritized in memory. The interplay between social and emotional factors is an interesting direction for future work, particularly in terms of the shared versus distinct effects of aging (Kensinger & Gutchess, 2015, 2017). The current study adds to evidence of at least some separation between these domains. Moreover, it will be important to determine which aspects of socioemotional information are most beneficial in memory or engage distinct systems. Although we have attempted to balance our positive and negative behaviors on dimensions such as arousal, it is possible they differ, or differ from neutral items, in other unintended ways. For example, distinctiveness could contribute (Hunt & McDaniel, 1993), as one is far more likely to make the acquaintance of someone who uses blue pens (a neutral item) than someone who is a murderer (negative item).

Our results have implications for the trait impression literature (e.g., Hess et al., 1998), which suggests that older adults would make less use of the specific information in forming their impressions and engage in less controlled processing, while the formation of an impression would be a relatively automatic process and less impaired with age. This is consistent with our findings of greater age differences in memory for specific information. Furthermore, when urged in Experiment 2 to rely more on “gut instinct” impressions formed through automatic processing and deliberation was discouraged, general memory performance was similar across age groups. Although differences across the two experiments prevent us from conclusively arguing that moving to more automatic processes better supported general memory performance, the pattern of reaction times is consistent with this suggestion. Reaction times were strikingly faster for Experiment 2 compared to Experiment 1, and disproportionately so for older adults. Although these experiments were not designed to be directly compared and several differences occur across them (e.g., for Experiment 2, participants decide amongst two alternatives vs. three, although are presented with two faces rather than one), the reaction time data nevertheless underscore the potential for older adults to benefit when impression memory can rely on automatic processes. Future work should further examine this question, employing process-specific tasks and tests of automaticity.

One consideration is that the nature of our memory tests may account for the diverging patterns of age effects for general and specific memory. The general memory tasks involved a forced-choice test with two or three options, while the specific memory task relied on recall. Recall is more difficult than recognition (e.g., Craik & McDowd, 1987; Davis, Trussell, & Klebe, 2001), so one concern is that the two types of memory we are measuring are more reflective of the differences in the tasks rather than differences in the types of memory. Although this should be addressed in future work, the diverging patterns of results across Experiments 1 and 2 are somewhat reassuring on this point. If the results simply reflected the greater difficulty of the specific memory test for older adults, then the pattern of age effects should be relatively similar across the two experiments. Despite the impairment to specific memory in both studies, the finding of age-equivalent general memory only occurs for Experiment 2, where there are supportive task conditions. In addition, specific memory performance is low in the experiments, despite attempts to reduce memory demands by providing multiple study trials, rich face and name cues at retrieval, and flexible scoring to capture the gist, or thematically-related information, rather than precise wording. Specific memory could also be lower in Experiment 2 due to the longer retrieval interval, and potential inference from the intervening general memory test. Ideally, the order of the tasks should be counterbalanced (although note that specific memory performance is still worse with age in Experiment 1, where the order did not differ). Relatedly, the age-equivalent general memory performance in Experiment 2 could reflect the demands of the task. Older adults can perform better on forced choice, rather than recognition tests, due to the contribution of familiarity (Bastin & Van der Linden, 2003), and age differences can track task difficulty such that they are larger for more difficult tasks (Earles & Kersten, 1998; Earles, Kersten, Berlin Mas, & Miccio, 2004). Although both of these factors could contribute to our findings of spared general memory, Experiment 1 also shared these features but did not identify age-equivalent effects. The contribution of these effects cannot be conclusively ruled out without further experimentation. At the very least, the present studies provide evidence that memory for general impressions can be intact with age, and highlight the types of conditions, including demands for specific recall versus consideration of more general impressions not reliant on explicit memory, that are important to explore further.

Despite these limitations, our study informs what is currently known about aging and cognitive change. According to Hess (1999), the creation of an impression relies on a mixture of both cognitive resources and processing goals, such as maintaining positive affect, but these goals are mediated by processing limits. Some of these processing limits that increase with age, such as declines in working memory and inhibitory ability, may lead to difficulty creating, changing, and accessing mental models (Radvansky, Zacks, & Hasher, 2005), such as the model one constructs when forming an impression. However, because both younger and older adults appear to process and use general evaluative information, such as that presented in our paradigm, in the same manner (Hess et al., 1998), we find that memory does not always simply reflect age differences in the ability to process information. General character information can be age-equivalent, even when older adults’ memory is lacking in specific details. These studies suggest that the distinction between measuring specific and general memory, taken into consideration with the demands and difficulty of the memory task, could be important in understanding when age differences in memory for socioemotional information do and do not emerge.

## Figures and Tables

**Table 1:** Demographic and neuropsychological measures (means and standard deviations) for younger and older adults in the two studies.

	Experiment 1			Experiment 2		

	Younger	Older	*p*-value	Younger	Older	*p*-value

Years Educ	13.20 *(1.15)*	15.95 *(2.40)*	<.001*	12.95 *(1.09)*	16.14 *(2.61)*	<.001*
Shipley Vocab	33.70 *(2.74)*	35.60 *(2.98)*	.04*	31.04 *(4.31)*	36.43 *(2.49)*	<.001*
Digit Comparison	70.85 *(10.13)*	55.55 *(9.63)*	<.001*	81.50 *(14.34)*	60.21 *(12.94)*	<.001*
Letter-Number Sequencing	12.40 *(1.98)*	10.00 *(2.22)*	.001*	12.14 *(2.41)*	9.61 *(2.36)*	<.001*
Verbal Paired Associates	25.50 *(4.42)*	19.15 *(8.76)*	.01*	25.46 *(6.36)*	16.39 *(8.74)*	<.001*
MMSE	n/a	29.05 *(1.10)*	n/a	n/a	28.43 *(1.26)*	n/a

**Table 2:** Reaction times for younger and older adults’ general memory decisions in Experiments 1 & 2.

**A. *Experiment 1***	**Positive**	**Neutral**	**Negative**

Younger	4382.46 *(254.59)*	5247.27 *(600.89)*	4855.18 *(511.66)*
Older	7889.50 *(787.54)*	11112.05*(1547.41)*	10829.10*(1728.04)*
**B. *Experiment 2***	**Positive vs. Negative**	**Positive vs. Neutral**	**Neutral vs. Negative**

Younger	2169.55 *(812.41)*	2413.80*(1071.99)*	2253.66*(1066.42)*
Older	4032.16*(2570.12)*	4515.61*(2722.81)*	4348.02*(2571.37)*

**Table 3:** Averages and Standard Deviations for General and Specific Memory Performance in Experiment 2.

	General Memory			Specific Memory		
	Neg/Neutral	Pos/Neg	Pos/Neutral	Positive	Neutral	Negative

**Younger**	.71 (.18)	.78 (.16)	.61 (.17)	.36 (.18)	.42 (.19)	.42 (.19)
**Older**	.78 (.19)	.80 (.19)	.63 (.17)	.21 (.16)	.27 (.20)	.24 (.15)

## Footnotes

1 One older adult mistakenly omitted birthdate from the demographics form, but was recruited from a sample that fell within this age range.

2 Initially each group consisted of 16 participants and preliminary analyses were conducted. Sample size was increased in response to reviewer suggestions.

3 We thank an anonymous reviewer for this suggestion.
